# Diverse target gene modifications in *Plasmodium falciparum* using Bxb1 integrase and an intronic *attB*

**DOI:** 10.1186/s13071-018-3129-5

**Published:** 2018-10-17

**Authors:** Praveen Balabaskaran-Nina, Sanjay A. Desai

**Affiliations:** 1 The Laboratory of Malaria and Vector Research, National Institute of Allergy and Infectious Diseases, National Institutes of Health, Rockville, MD 20852 USA; 2 Present Address: Department of Epidemiology and Public Health, Central University of Tamil Nadu, Thiruvarur, India

**Keywords:** Malaria, DNA transfection, Intron, Bxb1 integrase, Gene editing

## Abstract

Genetic manipulation of the human malaria parasite *Plasmodium falciparum* is needed to explore pathogen biology and evaluate antimalarial targets. It is, however, aggravated by a low transfection efficiency, a paucity of selectable markers and a biased A/T-rich genome. While various enabling technologies have been introduced over the past two decades, facile and broad-range modification of essential genes remains challenging. We recently devised a new application of the Bxb1 integrase strategy to meet this need through an intronic *attB* sequence within the gene of interest. Although this *attB* is silent and without effect on intron splicing or protein translation and function, it allows efficient gene modification with minimal risk of unwanted changes at other genomic sites. We describe the range of applications for this new method as well as specific cases where it is preferred over CRISPR-Cas9 and other technologies. The advantages and limitations of various strategies for endogenous gene editing are also discussed.

## Background

Despite advances from combination therapies and public health measures such as bednets, more than 400,000 people still die of malaria annually. Since *Plasmodium falciparum*, a virulent human malaria parasite, acquires resistance to most antimalarial drugs quickly, new drug targets and a better understanding of resistance mechanisms are needed to sustain advances in global health. These goals depend on transfection studies in cultured *P. falciparum* parasites [1–3]. Because transfection efficiencies are low, selectable markers are required; the available markers have facilitated functional analyses [4], but their relatively small number adds to the difficulties faced in molecular and biochemical studies of this pathogen.

Gene manipulation in this system requires single or double crossover homologous recombination into the haploid genome as the parasite lacks the enzymes to carryout non-homologous end joining. Historically, these experiments relied on infrequent, coincidental genome breaks at or near the target site, often necessitating many months of continuous cultivation and drug-cycling to obtain detectable levels of integration [5]. Further aggravating this process, the circular plasmids required for stable retention in transfected parasites tend to concatemerize under drug selection, reducing the likelihood of integration and complicating interpretation of some phenotypes [6, 7].

Subsequent implementation of *piggyBac* transposase and mycobacteriophage Bxb1 integrase facilitated stable integration of transgenes and reporters [8, 9], but these approaches have not been used to edit specific genes of interest. The advent of sequence-specific nucleases then opened the door to directed gene editing in *P. falciparum*, with zinc finger nucleases (ZFN) and clustered regulatory interspaced short palindromic repeats (CRISPRs) successfully used to study parasite biology [10–15]. Gene editing with ZFN is expensive and requires a new enzyme design for each target locus. As with many other organisms, CRISPR-Cas9 is quickly becoming the preferred strategy because of simplicity and cost, but several important concerns prevent the full spectrum of molecular modifications. Among these, off-target effects due to Cas9-mediated cleavage at unwanted genomic sites and the dependence on specific recognition motifs (known as protospacer adjacent motifs or PAMs) are considered the most problematic in many organisms [16].

To address these concerns and to add another option for DNA transfection in *P. falciparum*, we recently implemented a novel application of the Bxb1 integrase transposition technology to permit targeted gene replacements [17]. Our approach achieves rapid site-specific integration, allows the full spectrum of native gene modifications, and has a distinct set of advantages when compared to other methods including CRISPR-Cas9. Our strategy is based on the introduction of a silent *attB* element into an intron of the target gene; we found that this 40 bp insertion is well-tolerated as it does not adversely affect either intron splicing or subsequent translation and function of the encoded protein. Once integrated, this *attB* element permits rapid recombination with an *attP* site on subsequent transfection plasmids to replace the downstream sequence with desired modifications. Although our strategy requires an additional transfection to first introduce the *attB* site, it then enables an unlimited array of distal modifications including site-directed mutations, insertions, deletions, epitope tagging, and conditional knockdown through the introduction of ddFKBP or EcDHFR-based destabilization domains, the TetR aptamer module, or the *glmS* riboswitch [18–21]. After describing our procedures, we summarize the advantages and limitations of this approach when compared to currently available methods.

## Mechanism of *attB-attP* recombination and conventional application in *P. falciparum*

**Fig. 1:**
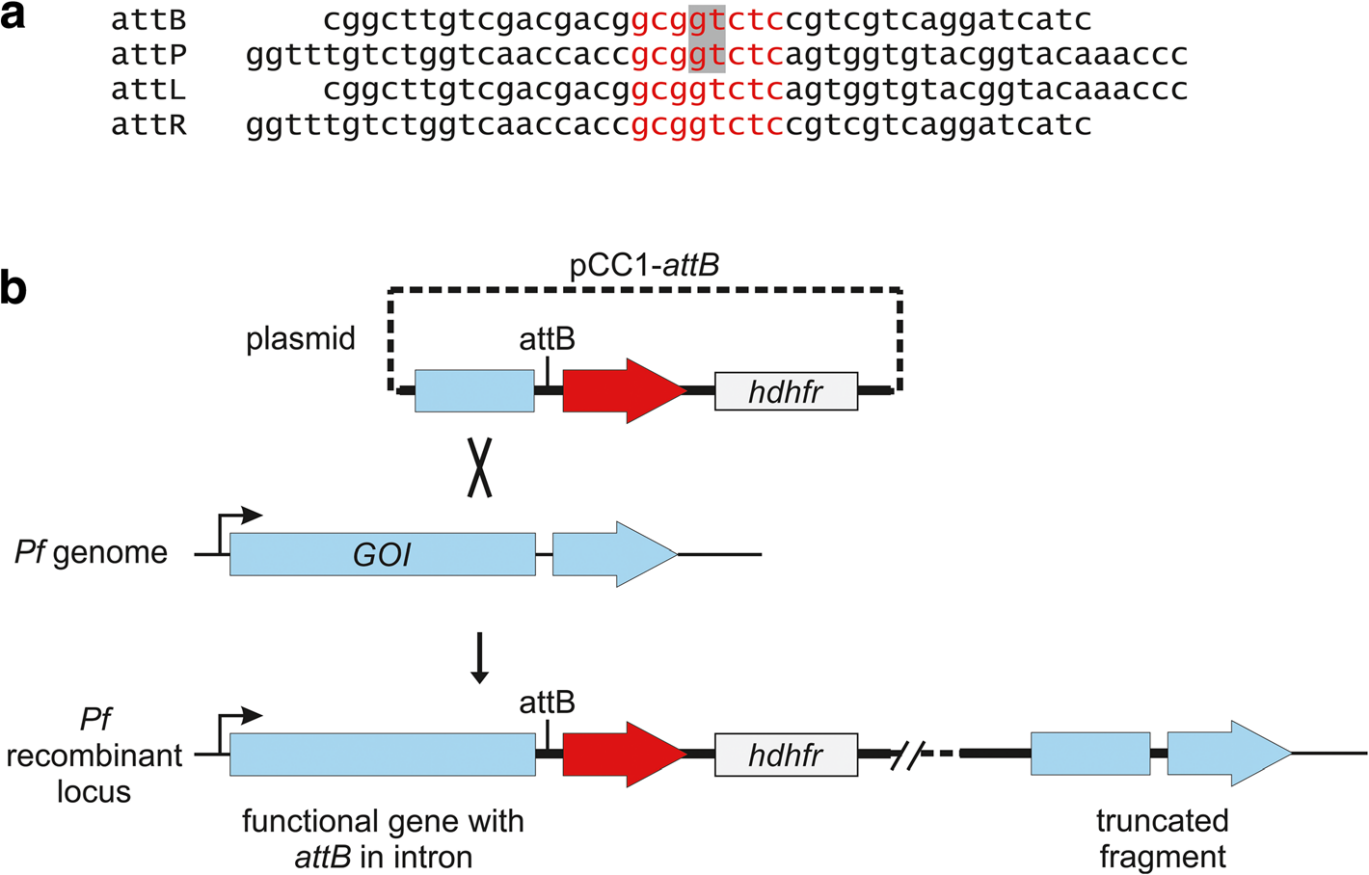
Introduction of silent *attB* element to enable gene editing. **a** Sequences of *attB* and *attP* used in *P. falciparum* transfections. Integrase-mediated recombination yields *attL* and *attR* elements as shown. The conserved 8 bp core sequence is shown in red; a non-palindromic 5’-GT dinucleotide (grey highlight) prevents self-ligation after cleavage by Bxb1 integrase and promotes efficient strand exchange. **b** Strategy for introducing *attB* into the intron of a gene of interest (GOI). The *pCC1-attB* plasmid carries an upstream sequence (light blue) to facilitate homologous recombination into the parasite genome, an intron with an inserted *attB*, and a recodonized version of the remainder of the gene (red). The *hdhfr* cassette permits selection of successfully transfected parasites. Recombination of the plasmid into the parasite genome yields the required *attB*-carrying parasite without affecting target gene transcription or associated phenotypes

Bxb1 integrase is a mycobacteriophage serine integrase that catalyses recombination between an *attP* sequence in the phage and an *attB* sequence in the *Mycobacterium smegmatis groEL1* gene with minimal fully effective sequences of 48 and 38 bp, respectively. This recombination enables phage integration into the bacterial genome [22–24]*.* Recombination begins when Bxb1 dimers bind to each of these sites with high affinity (*K*_*d*_ estimated at 70 nM); these dimers then interact with each other to form a synaptic tetramer, a process known as synapsis [25]. All four DNA strands are then cleaved within a central 8 bp core conserved between *attB* and *attP* (Fig. 1a); cleavage occurs asymmetrically at a non-palindromic 5’-GT dinucleotide to ensure faithful exchange of phage *attP* and bacterial *attB* sequences and prevent incorrect joining of half-sites [24]. A conserved serine in Bxb1 forms a covalent bond with the cleaved DNA, allowing rotation and re-ligation of the two double-stranded DNA polymers. The resulting sites, termed *attL* and *attR*, differ from one another and do not match the original *attB* and *attP* elements as they carry swapped flanking sequences. This reaction is generally considered irreversible, but auxiliary proteins known as the recombination directionality factors (RDFs) can facilitate the reverse reaction and excision of the phage from the host genome [26]. Structural studies have revealed extensive contacts between the recombinase domain, an unusual zinc ribbon domain, and *att* sequences that confer specificity for *attB-attP* recombination and prevent the reverse *attL*-*attR* recombination reaction [27]. Kinetic and mutagenesis experiments have led to a model of highly coordinated formation of a synaptic complex between a tetrameric recombinase complex and two *att* sites, controlled cleavage and rotation, hybridization and religation [28–31]. When compared to tyrosine integrases, serine integrases do not require DNA super coiling, divalent cations or bacterial host factors for integrase activity, making their use in heterologous systems more attractive.

Nkrumah et al. [9] demonstrated this system’s utility in malaria parasites by showing efficient recombination of an *attP-*containing plasmid with an engineered *attB* in the parasite genome; Bxb1 integrase was expressed from a co-transfected helper plasmid. The *attB* sequence, otherwise not present in *P. falciparum*, was introduced into the nonessential *cg6* gene; several such *cg6-attB* lines have been generated in distinct genetic backgrounds [32]. These studies reported a rapid appearance of recombinants with a near-homogeneous population of integrant parasites within 2–4 weeks of the second transfection [9]. Nevertheless, several concerns have limited the utility of this technology. Most importantly, in cases where the desired transgene on the *attP* plasmid is a modified version of a parasite gene, the endogenous copy is not affected by transfection and is expected to remain functional, yielding a merodiploid whose phenotype may be more difficult to study. Expression levels for the transgene may also confound interpretation: genomic site effects due to expression from a heterologous *cg6* locus or concatemerization of the *attP* plasmid prior to integration may lead to unanticipated transcript levels. Another concern is that the phenotype under study may be affected by disruption of the *cg6* gene. Production of lines that carry *attB* at nonessential loci other than *cg6* could address some of these concerns, but this has not been undertaken to our knowledge.

While Cre recombinase, a distinct phage recombinase from the tyrosine integrase family [33], has been used in various parasites [34–36], use of Bxb1 integrase has not been described for Apicomplexan parasites other than *Plasmodium* spp.

## Integrase strategy for native gene manipulation in *Plasmodium falciparum*

To address these limitations and enable rapid and essentially unlimited manipulation of an endogenous parasite gene, we devised and used a modified version of this Bxb1 integrase strategy [17]. Our approach entails introducing *attB* into an intron of the gene of interest (Fig. 1b); we found that this relatively short element did not adversely affect mRNA splicing, transcription or translation of *clag3*, an intensively studied gene linked to malaria parasite nutrient uptake [37–39]. As changes to the intronic sequence are silent, the encoded protein should be faithfully translated without mutation or changes in associated phenotypes. Consistent with this prediction, our studies with the *clag3* target revealed expression of a full-length unmodified protein, unchanged trafficking of the encoded protein to the host membrane, and preserved channel-mediated solute uptake and pharmacology [17]. As knockdown or modification of the *clag3* gene product is associated with a significant fitness cost [40, 41], these findings suggest that most essential genes will tolerate addition of an intronic *attB* sequence.

We envision that this approach will be broadly applicable to study of *P. falciparum* genes. Approximately half of this parasite’s genes have one or more introns with a small average size, 179 bp [42], simplifying addition of *attB* and *attP* sequences. This cloning step can be performed either through DNA synthesis or through hybridization of complementary oligonucleotides in cases where the introns are small. Consistent with previous additions of short sequences to this parasite’s introns [43], these small silent insertions are generally well-tolerated in *P. falciparum*. Although we have not examined the effect of position within the intron systematically, we suggest a central placement within the intron and preferably at least 60 nucleotides upstream of the 3’ end of the intron as splicing branch points have been observed up to 57 nucleotides from the 3’ intron-exon boundary [42]. For genes that lack introns, insertion of a heterologous intron carrying the *attB* sequence into the open reading frame (ORF) offers excellent prospects for introduction of a silent *attB* without requiring modifying the encoded protein’s sequence; our *clag3 attB* intron has been validated and should work in most cases based on studies confirming faithful recognition and splicing of heterologous introns [43].

We originally introduced the *attB*-loaded intron into *clag3 via* single crossover recombination, but this may be simplified by CRISPR-Cas9 transfection with a donor plasmid that contains homology arms of 200–300 bp on either side of the intron; if CRISPR-Cas9 is used, it is critical to select the single guide RNA (sgRNA) and cleavage site carefully with proximity to the intron and on-target efficiency score the most critical factors in *P. falciparum* transfections [44]. After parasites grow out in this first transfection, it is important to use limiting dilution cloning to obtain a clonal population carrying the *attB* sequence. Although some workers have argued that limiting dilution cloning is not required because subpopulations without this integration event will be removed by cloning after the second transfection, we caution against this because genome-level modifications at non-target sites may grow preferentially in the second transfection, aggravating attempts to identify the desired final clone.

**Fig. 2:**
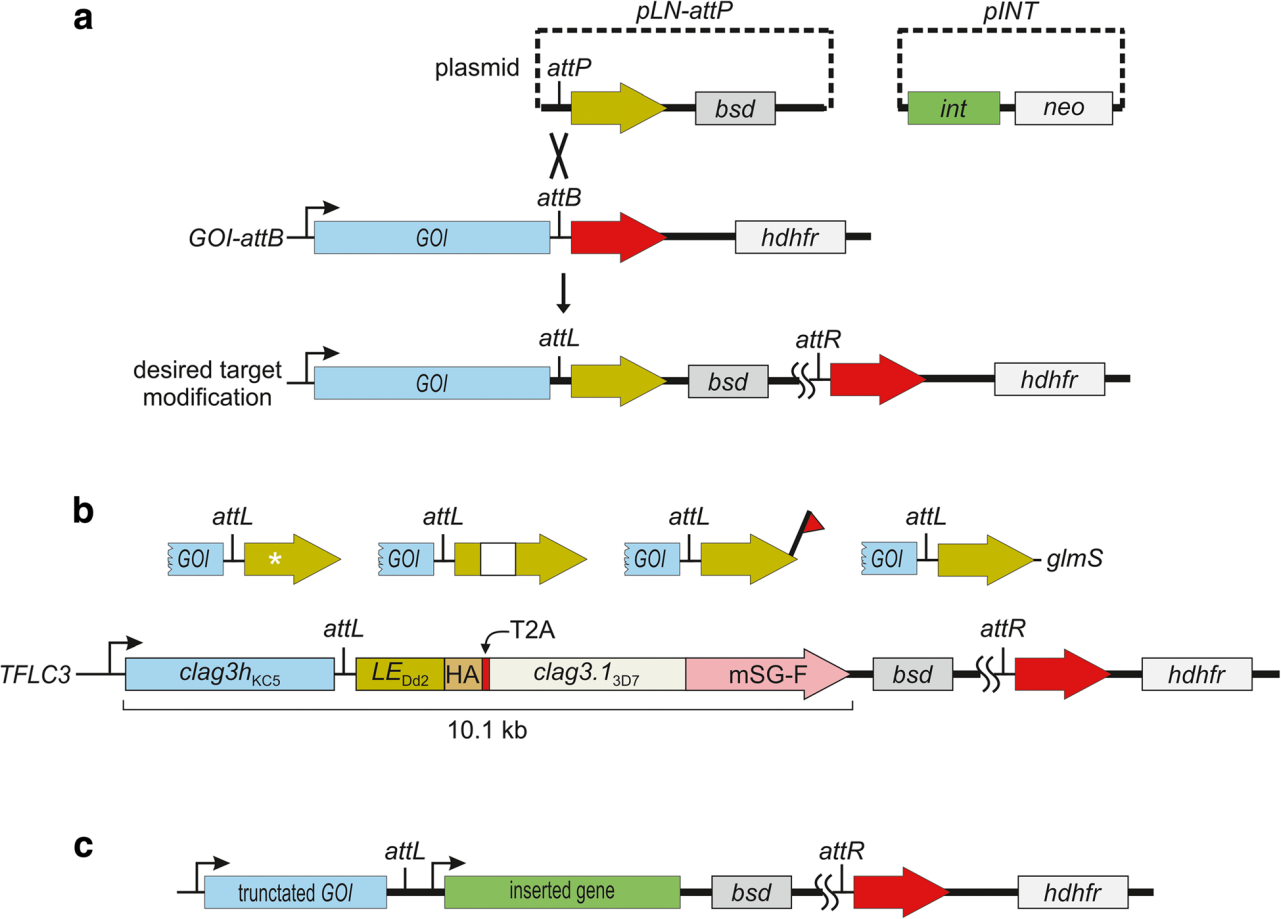
A broad range of gene modifications using the intronic *attB*. **a** Strategy for Bxb1 integrase-mediated recombination to produce diverse changes to a target gene. The *pLN-attP* plasmid carries the *attP* element, the downstream intronic sequence and desired downstream modifications. *bsd*, *neo* and *hdhfr* are selectable markers; *int* represents Bxb1 integrase gene as expressed from the *pINT* helper plasmid. Transfection of the *GOI-attB* parasite replaces the downstream ORF (red arrow) with a desired modification (yellow arrow) of the gene of interest (*GOI*). Recombination between *attB* and *attP* elements yields *attL* and *attR* sites. **b** Example modifications. Top row shows a site-directed mutation, an insertion, an epitope tag, and the *glmS* riboswitch in the 3’ untranslated region (left to right, respectively). Second row shows production of the *TFLC3* parasite expressing two full-length CLAG3 proteins (Dd2 and 3D7 isoforms) with distinct epitope tags (HA and mSG-F) under a single *clag3* promoter; the two proteins are separated during translation through insertion of a viral skip peptide (T2A, [52]). **c** Gene replacement approach showing insertion of a full-length gene with promoter and terminator sequences after the silent *attB*. This approach simultaneously disrupts the *GOI* and integrates a cloned gene downstream of the target site

After the silent *attB* site has been successfully introduced, the full range of target gene modifications can be generated with a second transfection with two plasmids (Fig. 2a, pLN-*attP* and pINT). The pLN-*attP* plasmid carries the *attP* element followed by the distal intronic sequence with a 3’ splice site for faithful splicing after integration. Any sequence following this *attP*-intron will be inserted into the genome upon *attB-attP* recombination as facilitated by the Bxb1 integrase expressed from the pINT helper plasmid.

The use of a silent *attB* in a target gene’s intron distinguishes our approach from previous use of this technology in malaria research and has two important consequences. It disrupts the target gene by inserting the entire pLN-*attP* plasmid in the gene after the modified intron. It also achieves a promoter-trap because the sequence inserted distal to the intron upon recombination will be spliced in frame behind the native promoter and gene sequence upstream of the intron. Thus, the outcome in most cases is an effective gene replacement. In one application, this distal sequence could be the remainder of the gene with desired modifications. These modifications may include site-directed mutations, internal insertions or deletions (indels), domain swaps, addition of C-terminal epitope tags on the encoded protein, modified codon usage, and/or altered transcriptional regulatory elements (e.g. removal of distal introns or addition of the *glmS* riboswitch into the 3’ untranslated region). To demonstrate the range of possible modifications, we reported the production of a merodiploid parasite that expresses two CLAG3 proteins with distinct functional properties and separate epitope tags using this approach; although these *clag3* genes are large (5.3 kb each) and we desired distinct epitope tags on two proteins expressed under a single promoter, our strategy and use of the *clag3-attB* line permitted facile introduction with a single plasmid (Fig. 2b, *TFLC3*; [17]).

In another application, the promoter trap enabled by the silent *attB*-intron is disregarded, and the pLN-*attP* plasmid may carry an entire gene cassette with required 5’ and 3’ untranslated regions (Fig. 2c). This may be particularly useful for examining whether other genes (e.g. paralogs) can adequately replace essential activities of the target gene carrying the *attB*-intron. For such applications, an important caveat is that the sequence upstream of the intron will typically still be transcribed and translated, yielding a truncated protein. For large genes such as *clag3*, the effect of the residual upstream protein may be significant, yielding either preserved activity or a dominant-negative effect due to protein-protein interactions with the truncated protein. This theoretical concern may be largely avoided by introducing the *attB* into an intron near the start of the target gene. If such an intron is not available, a heterologous *attB-*containing intron may be inserted at any desired position as described above.

## Comparison to other methods available for targeted gene modifications

While there are several methods for expression of transgenes in *P. falciparum* [45], there are only a few options for directed modification of a target gene of interest. Table 1 summarizes these options along with the required components, advantages and limitations of each method. Although CRISPR-Cas9 is the most straightforward to implement and allows rapid gene editing suitable for most applications [11, 12, 44], use of Bxb1 integrase with an engineered intronic *attB* site may be preferred for large insertions and has several other advantages.

**Table 1:** Strategies for target gene modification in *P. falciparum*

Method	Mechanism	Required elements	Advantages	Key limitations
Single or double crossover (with or without SLI)	Homologous recombination between a targeting sequence on transfection plasmid and the gene of interest	1 or 2 large homology arms on the plasmid	(i) Does not require expression of heterologous nucleases or enzymes; (ii) The foundation of allelic replacement strategies in malaria research	(i) Depends on coincidental genome breaks near the target; (ii) Cannot prevent gradual loss of the desired modification due to plasmid loop-out (avoided by SLI); (iii) Requires months, usually with rounds of drug cycling to select for genomic integration (reduced with SLI)
Zinc-finger nuclease (ZFN)	Engineered ZFN produces a double-stranded break, which is repaired by homologous recombination with plasmid	(i) Custom engineered ZFN for each target site; (ii) 2 homology arms for HDR, typically on a separate plasmid	(i) Rapid gene editing; (ii) No sequence-specific restrictions such as PAM; (iii) Longer track-record of use than CRISPR-Cas9, including clinical trials in humans	(i) Expensive; (ii) Requires custom ZFN production for each target site; (iii) Off-target nuclease activity may be comparable to CRISPR-Cas9
CRISPR-Cas9	sgRNA directs Cas9 nuclease to produce a double-stranded break, which is repaired by homologous recombination with plasmid	(i) Cas9 nuclease; (ii) sgRNA expressed under a U6 promoter or by heterologous T7 polymerase; (iii) 2 homology arms for HDR, typically on a separate plasmid	(i) Rapid gene editing (ii) Easy to implement in most labs	(i) Cleavage limited to sites adjacent to PAM sequence; (ii) Requires careful consideration of on-target efficiency score for optimal design; (iii) Off-target cleavage
Bxb1 integrase and intronic *attB*	*attB* introduced into target gene intron (or via an engineered synthetic intron) undergoes specific recombination with *attP* element on transfection plasmid, facilitated by Bxb1 integrase	(i) Intronic *attB*; (ii) Bxb1 integrase; (iii) Desired gene modification is placed distal to *attP* on plasmid	(i) Rapid gene editing; (ii) Allows diverse modifications on a gene of interest; (iii) Does not depend on HDR, so avoids cloning of AT-rich homology arms; (iv) Little or no risk of off-target recombination; (v) Permits larger target site insertions than CRISPR-Cas9	(i) Requires production of cloned parasite with intronic *attB* for each gene of interest; (ii) Location of intronic *attB* within the target gene must be carefully selected to enable desired gene modifications

Our use of an intronic *attB* site resembles the recent description of *loxP* insertion into introns for conditional editing of a target gene [43], but there are important differences that confer greater versatility to *attB*-*attP* technology. Both use a target gene intron to introduce short DNA elements that enable enzyme-mediated DNA recombination. The DiCre-*loxP* system sites uses two halves of Cre recombinase linked to distinct rapamycin binding proteins; addition of rapamycin stimulates dimerization of the two halves, allowing conditional Cre activity and recombination at two properly oriented *loxP* sites. This system permits conditional knockout or modification such as addition of an epitope tag or site-directed mutation [43]. The main strength of this system relative to our *attB-*intron system is that it allows conditional modifications dependent on small molecule addition. At the same time, a key limitation is that facilitated recombination between the two *loxP* sites is reversible, in contrast to irreversible recombination between *attB* and *attP* sites [26]. This reversibility prevents use of a single initial transfectant with a *loxP*-intron for multiple distinct modifications: a plasmid carrying a *loxP* with desired distal modifications, analogous to our pLN-*attP* plasmid (Fig. 2a), would likely undergo cycles of integration and excision due to the reversibility of *loxP* recombination. This results in an inefficient integration reaction; although various strategies have been devised to overcome this inefficiency [46], these have not been implemented in malaria research to our knowledge. Thus, multiple distinct modifications cannot be performed with a single engineered *loxP* parasite: each desired modification requires a *de novo* transfection to introduce the *loxP*-intron cassette into the parasite genome.

As increasingly acknowledged by workers, CRISPR-Cas9 technology will be the method of choice for most gene modifications in *P. falciparum*. This system largely bypasses the need for introduction of DNA motifs such as *attB* or *attP*; it requires expression of Cas9, an RNA-guided endonuclease, and a small single-stranded RNA molecule known as the single guide RNA or sgRNA to achieve site-specific modification of the genome [47]. The sgRNA binds Cas9 and identifies the cleavage site through Watson-Crick base pairing over a 20 bp recognition sequence; gene editing results from blunt-end cleavage within the recognition sequence. Although a protospacer adjacent domain (PAM) must be present immediately after the chosen recognition sequence, there are nearly 663,000 canonical PAM sites in the *P. falciparum* genome [44]; thus, almost any genomic site can be targeted. Nevertheless, we considered two important cases where CRISPR-Cas9 may not be the ideal method for target gene modifications. One problematic situation arises when a gene of interest is a member of a multigene family. If the site to be modified in the target gene is conserved amongst paralogs that should not be edited, it may be difficult to find an sgRNA that does not cleave one or more of these paralogs; such “off-target” cleavage sites may be predicted and avoided with a newly devised paralog specificity score [44]. If multiple modifications of a target gene are envisioned, use of the CRISPR system may still require greater effort and cost because confident production of each modification will require screening of limiting dilution clones to identify parasites with the desired target gene modification and no unwanted edits to paralogs. The *attB-*intron strategy reduces labor and expense because the identification of a clone without unwanted *attB* introduction into paralogs need only be done once: subsequent transfections with *attP-*containing plasmids will then be highly specific for the desired target gene, confidently avoiding paralogs.

Even for genes not within multigene families, off-target effects remain a concern with CRISPR-Cas9 editing because the Cas9 nuclease can recognize and cleave at sites carrying one or more mismatches relative to the selected 20 bp recognition sequence. This limitation has led to the development of predictive off-target scoring algorithms and Cas9 mutants with ongoing improvements in specificity [16, 48]. The targeted genome break produced by Cas9 nuclease may also lead to chromosome repair by production of large deletions or more complex rearrangements [49], although these have not been reported to date in malaria research. Promiscuous recombination and target site rearrangements have not been reported with the Bxb1 integrase, presumably because of the highly coordinated *attB*-*attP* recombination process [50].

Another important case where the *attB*-intron strategy will be preferred over technologies such as CRISPR-Cas9 and selection-linked integration (SLI, [51]) is exemplified by the production of a parasite carrying two *clag3* genes under the native promoter (*TLFC3* parasite in Fig. 2b). SLI selects specifically for homologous recombination at the target site by using a promoter trap to drive expression of the selectable marker gene. Because both CRISPR-Cas9 and SLI depend on homologous recombination, both would require fully re-codonized versions of the two *clag3* genes to produce *TFLC3* to avoid internal recombination events that bypass introduction of desired tags on the two *clag3* alleles (“HA” and “mSG-F” in Fig. 2b *TFLC3* ribbon). This re-codonization would require custom synthesis of a 6.3 kb DNA, which is both prohibitively expensive and time-consuming for most research labs.

## Conclusions

DNA transfection technologies represent an important tool for basic and translational malaria research. We remain limited by a low transfection efficiency, relatively slow parasite replication, a paucity of selection markers, and other difficulties specific to this important pathogen. Ongoing improvements in the methods available for generating parasite lines with desired genome modifications make this is an exciting time for malaria research. While CRISPR-Cas9 is currently the method-of-choice for most gene editing experiments, other strategies such as our *attB-*intron method should be considered, especially for labs that have committed to specific genes and require a broad collection of gene modifications for biochemical and/or structural studies.

## Abbreviations

*Bsd*: Blasticidin S deaminase selectable marker
Cas9: CRISPR associated protein 9
*Clag3*: Cytoadherence linked asexual gene 3
CRISPR: Clustered regularly interspaced short palindromic repeats
GOI: Gene of interest
*hdhfr*: Human dihydrofolate reductase selectable marker
HDR: Homology directed repair
indels: Internal insertions or deletions
*neo*: Neomycin selectable marker
ORF: Open reading frame
PAM: Protospacer adjacent motif
sgRNA: Single guide RNA
SLI: Selection linked integration
*TFLC3*: Tandem full-length *clag3* parasite
ZFN: Zinc finger nuclease
